# Supplementary material for: Association between XRCC3 p.Thr241Met polymorphism and risk of glioma: A systematic review and meta-analysis
Source: PLoS One. 2022 Oct 20;17(10):e0276313. doi: 10.1371/journal.pone.0276313 (PMC9584405; doi:10.1371/journal.pone.0276313)
Supplement: S1 Table — (DOCX) [file pone.0276313.s001.docx]

**S1 Table: Quality assessment of the included studies**

| **Study ID ^reference^** | **Selection** | | | | **Comparability** | **Exposure** | | | **Total star** |
| --- | --- | --- | --- | --- | --- | --- | --- | --- | --- |
|  | **Criterion 1** | **Criterion 2** | **Criterion 3** | **Criterion 4** | **Criterion 1** | **Criterion 1** | **Criterion 2** | **Criterion 3** |  |
| Huang 2015 (32) | ★ | ★ |  | ★ | ★ |  | ★ | ★ | 6 |
| Gao 2014 (33) | ★ | ★ |  | ★ | ★★ | ★ | ★ | ★ | 8 |
| Rodriguez-Hernandez 2014 (34) | ★ | ★ |  | ★ | ★ |  | ★ | ★ | 6 |
| Xu 2014 (35) | ★ | ★ |  | ★ | ★★ |  | ★ | ★ | 7 |
| Luo 2013 (36) |  | ★ |  | ★ | ★ |  | ★ | ★ | 5 |
| Pan 2013 (37) | ★ | ★ |  | ★ | ★ | ★ | ★ | ★ | 7 |
| Zhao 2013 (38) | ★ | ★ |  | ★ | ★★ | ★ | ★ | ★ | 8 |
| Custódio 2012 (39) | ★ | ★ |  | ★ | ★ |  | ★ | ★ | 6 |
| Liu 2012 (40) | ★ | ★ |  | ★ | ★ |  | ★ | ★ | 6 |
| Rajaraman 2010 (22) | ★ | ★ |  | ★ | ★ |  | ★ | ★ | 6 |
| Liu 2009 (41) | ★ | ★ | ★ | ★ | ★★ |  | ★ | ★ | 8 |
| Zhou 2009 (42) | ★ | ★ |  | ★ | ★★ | ★ | ★ |  | 7 |
| Kiuru 2008 (16) | ★ | ★ |  | ★ | ★★ |  | ★ | ★ | 7 |
| Wang 2004 (19) | ★ | ★ |  | ★ | ★★ |  | ★ | ★ | 7 |
