# Supplementary figures and images for: Association between XRCC3 p.Thr241Met polymorphism and risk of glioma: A systematic review and meta-analysis
Source: PLoS One. 2022 Oct 20;17(10):e0276313. doi: 10.1371/journal.pone.0276313 (PMC9584405; doi:10.1371/journal.pone.0276313)

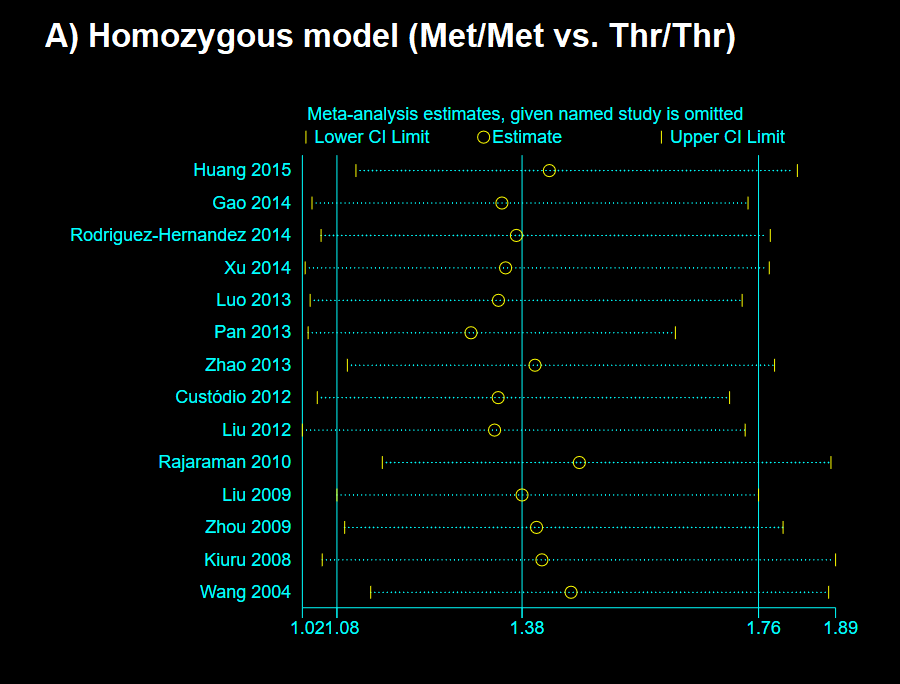

Supplement: S1 Fig — (ZIP) [file pone.0276313.s002.zip › S1a_Fig.tif]

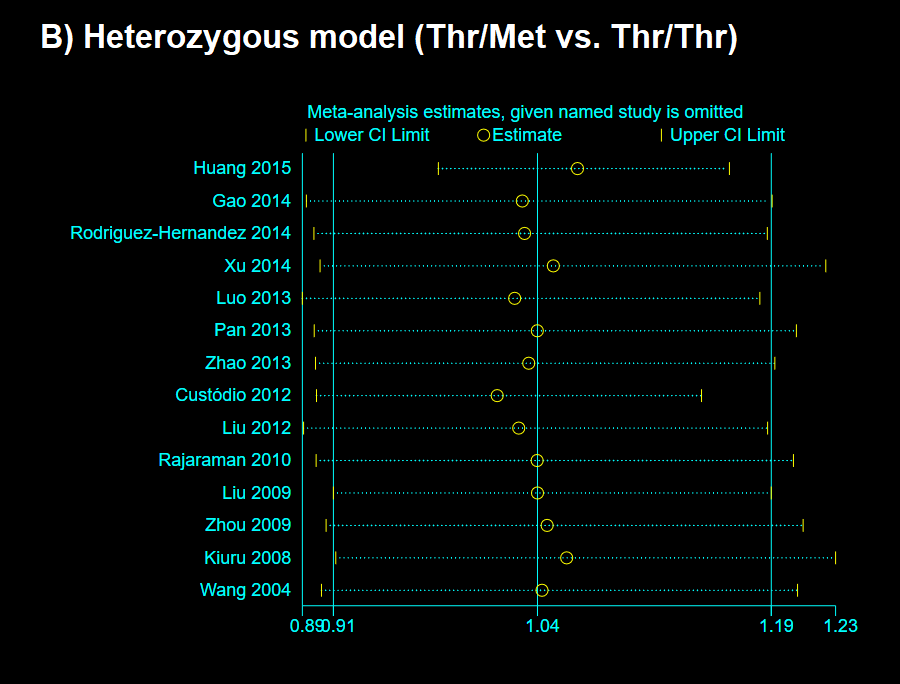

Supplement: S1 Fig — (ZIP) [file pone.0276313.s002.zip › S1b_Fig.tif]

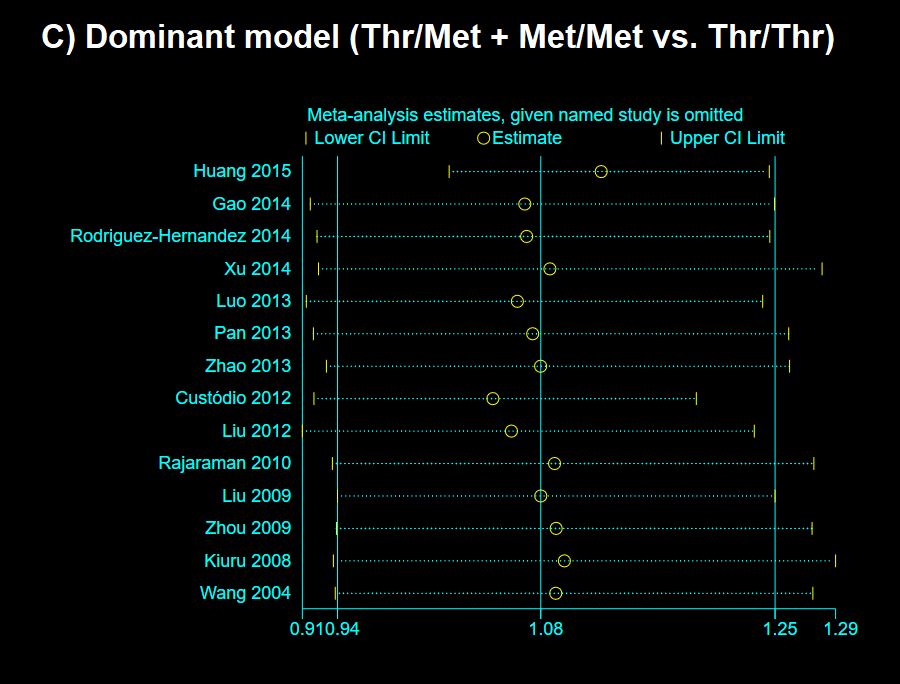

Supplement: S1 Fig — (ZIP) [file pone.0276313.s002.zip › S1c_Fig.tif]

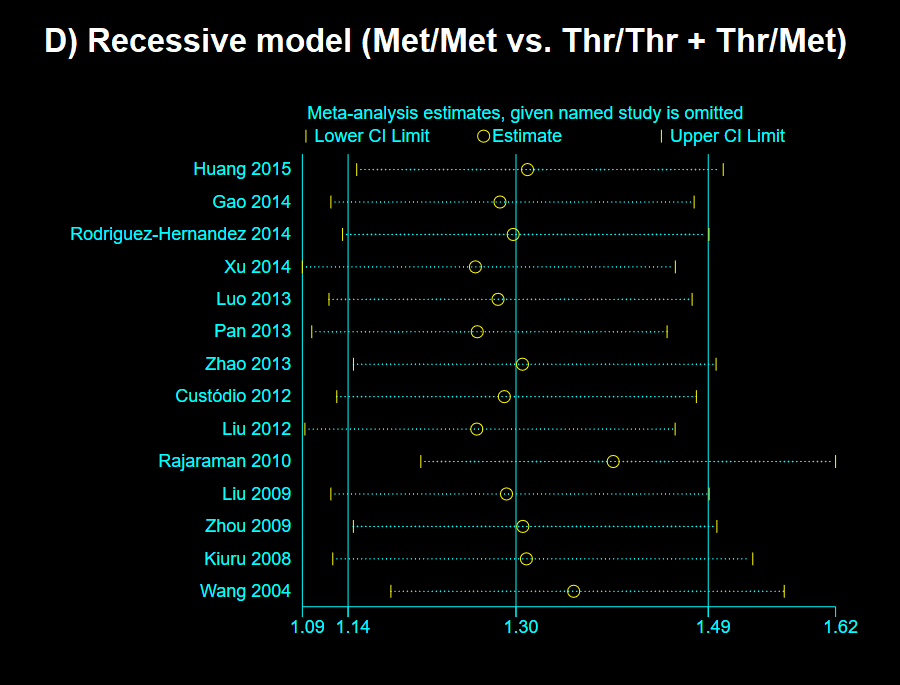

Supplement: S1 Fig — (ZIP) [file pone.0276313.s002.zip › S1d_Fig.tif]

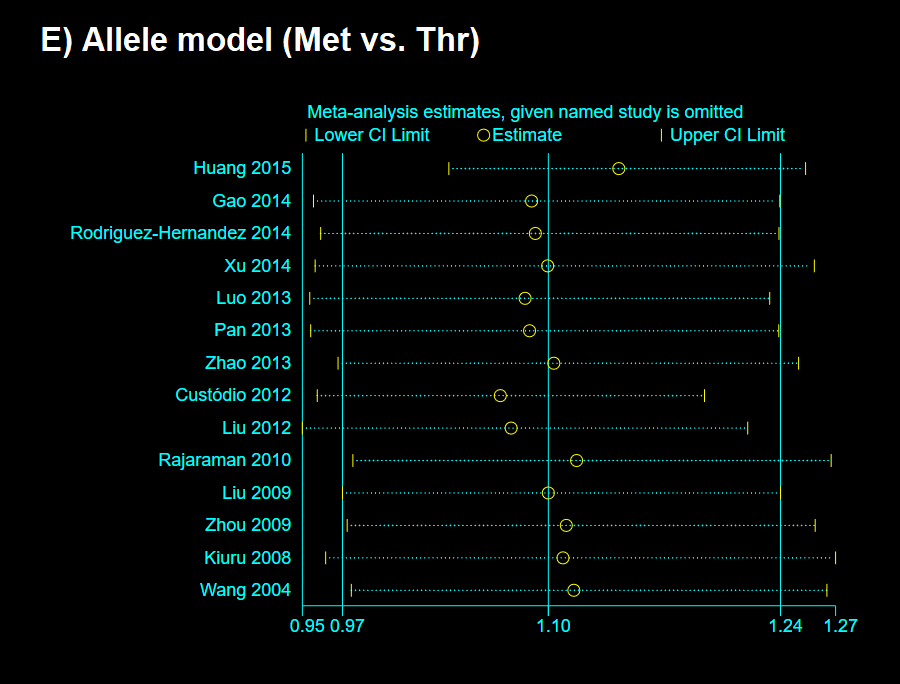

Supplement: S1 Fig — (ZIP) [file pone.0276313.s002.zip › S1e_Fig.tif]
