# Supplementary material for: Association between XRCC3 p.Thr241Met polymorphism and risk of glioma: A systematic review and meta-analysis
Source: PLoS One. 2022 Oct 20;17(10):e0276313. doi: 10.1371/journal.pone.0276313 (PMC9584405; doi:10.1371/journal.pone.0276313)
Supplement: S1 File — (DOCX) [file pone.0276313.s003.docx]

**S1 File**

**Modified Newcastle-Ottawa scale for meta-analysis of genetic association studies**

A study can receive a maximum of one star for each numbered item within the Selection and Exposure categories. A maximum of two stars can be awarded for Comparability.

Selection

1. Appropriateness of cases
2. Diagnosis was done with independent validation (e.g. histopathological examination) *
3. Diagnosis was based on unreliable methods (e.g. self-reports)
4. Case definition was unclear or not available
5. Representativeness of cases
6. Cases were obviously representative of the population studied *
7. There was potential for selection bias
8. Selection of controls
9. Controls were population-based AND genetic polymorphism of interest conformed to HWE *
10. Controls were not recruited from the general population (e.g. hospital-based controls)
11. Genetic polymorphism of interest did not conform to HWE
12. Appropriateness of controls
13. Controls were defined as having no history of disease under studied AND was appropriate for the condition studied *
14. Inappropriate controls were used (e.g. used male controls for studies of female breast cancer)
15. Control definition was unclear or not available

Comparability

1. Comparability of cases and controls on the basis of the design or analysis
2. Cases and controls were of homogeneous ethnic descent *
3. There was no evidence of population stratification *
4. Comparability of cases and controls was unknown

Exposure

1. Ascertainment of exposure
2. Quality control procedures was applied to validate the genotype AND the personnel was blinded to phenotype status during genotyping *
3. Quality control procedures was applied but phenotype status was not blinded
4. Quality control procedures or blinding status was not unknown
5. Same method of ascertainment for cases and controls
6. Yes *
7. No
8. Genotyping call rate
9. ≥ 99% call rate *
10. < 99% or unknown call rate
